# Neuronal sphingosine kinase 2 subcellular localization is altered in Alzheimer’s disease brain

**DOI:** 10.1186/s40478-018-0527-z

**Published:** 2018-04-03

**Authors:** Gaëlle Dominguez, Marie-Lise Maddelein, Mélanie Pucelle, Yvan Nicaise, Claude-Alain Maurage, Charles Duyckaerts, Olivier Cuvillier, Marie-Bernadette Delisle

**Affiliations:** 1 Université de Toulouse, Inserm UMR 1214, 31000 Toulouse, France; 2 Institut de Pharmacologie et de Biologie Structurale, Université de Toulouse, CNRS, UPS, Toulouse, France; 3 CHU de Toulouse, Laboratoire de Neuropathologie, 31059 Toulouse, France; 4 Université de Toulouse III, Faculté de Médecine Rangueil, 31062 Toulouse, France; 5 Université Lille, Inserm, UMR 837, 59000 Lille, France; 6 CHU Lille, Service de Pathologie, 59037 Lille, France; 7 Laboratoire de Neuropathologie et Centre de Recherche de l’ICM, Hôpital de la Salpétrière, 75013 Paris, France

**Keywords:** Alzheimer’s disease, Neuropathology, β-amyloid, Sphingolipids, Sphingosine kinase 2, Sphingosine 1-phosphate

## Abstract

**Background:**

Alzheimer’s disease (AD) is characterized by the accumulation of β-amyloid (Aβ) peptides and hyperphosphorylated tau protein accompanied by neuronal loss. Aβ accumulation has been associated with an impaired sphingosine 1-phosphate (S1P) metabolism. S1P is generated by sphingosine kinases (SphKs), of which there are two isoenzymes SphK1 and SphK2, and degraded by the sphingosine 1-phosphate lyase (SPL). We previously reported, that both a decrease in SphK1 expression and an increase in SPL expression, correlated with amyloid deposits in the entorhinal cortex of AD brains, suggesting a global loss of pro-survival S1P in AD neurons. SphK2 contribution has also been examined in AD yielding to conflicting results that may reflect the complexity of SphK2 regulation. The subcellular localization of SphK2, hence the compartmentalization of generated S1P, is recognized to play a crucial role in dictating either its pro-survival or pro-apoptotic functions. We therefore aimed at studying the expression of SphK2 and notably its subcellular localization in brain tissues from patients with AD.

**Results:**

We report that a decrease in SphK2 protein cytosolic expression correlated with the density of amyloid deposits in a cohort of 25 post-mortem brains. Interestingly, we observed that the equilibrium between cytoplasmic and nuclear SphK2 is disrupted and showed that SphK2 is preferentially localized in the nucleus in AD brain extracts as compared to control extracts, with a marked increase of cleaved SphK2.

**Conclusions:**

Our results suggest that a shift in the subcellular localization of the S1P generating SphK2 may compromise the well established pro-survival cytosolic S1P by favoring the production of nuclear S1P associated with adverse effects in AD pathogenesis.

**Electronic supplementary material:**

The online version of this article (10.1186/s40478-018-0527-z) contains supplementary material, which is available to authorized users.

## Introduction

Alzheimer’s disease (AD) currently affects over 46 million people in the world and this number is estimated to reach 73 million by 2030 [54]. Gaining a better understanding of the disease is therefore critical to develop efficient treatments. AD is characterized by two paramount lesions: i) intracellular accumulation of hyperphosphorylated Tau protein forming neurofibrillary tangles (NFT) and neuropil threads; and ii) extracellular deposits of β-amyloid (Aβ) peptide, leading to both diffuse and neuritic plaques. These lesions are associated with neuronal loss, inflammatory process and synaptic dysfunction [16, 17]. The regions affected by both lesions are hierarchically involved. Initially confined to specific locations, frontal and entorhinal cortex, hippocampus, the lesions progressively involve most areas of the brain defining stages in the disease [6, 50].

Sphingolipids are ubiquitous lipid components of membranes that are metabolized to form signaling molecules associated with cellular activities important for health and disease. Evidences are accumulating to argue on their key role in neurodegeneration [9, 28]. One of the most important of these metabolites is sphingosine 1-phosphate (S1P), which is involved in the onset or the progression of pathological conditions including cancer, autoimmunity, cardiovascular conditions or diabetes [37]. The S1P content in cells is low and is kept under control through a delicately regulated balance between its synthesis and its degradation. The balance between the intracellular levels of S1P and its metabolic precursors, ceramide and sphingosine, has been suggested to be a switch determining whether a cell proliferates or dies [14]. S1P is generated by sphingosine kinases (SphKs), of which there are two isoenzymes (SphK1 and SphK2), and is degraded by the S1P lyase (SPL) to hexadecenal and ethanolamine phosphate [44]. Once produced, S1P can work as an intracellular signaling molecule or be secreted to act as an autocrine or paracrine molecule by binding to five specific high-affinity G protein-coupled receptors (GPCR), named S1P 1–5 [13, 45, 46]. Subcellular localization of SphK1 and SphK2 isoenzymes and subsequent compartimentalization of generated S1P appear to be crucial in dictating the biological effect of S1P [40]. It is well established that in order for SphK1 to mediate pro-proliferative and pro-survival signaling, it must relocalize from the cytoplasm to the plasma membrane. In contrast, the subcellular localization of SphK2 appears to be much more complex, like its biological effects [40]. For instance, when present in the nuclei, SphK2 has been associated with DNA synthesis inhibition [27] or HDAC regulation [24]. In the mitochondria, SphK2 cooperates with Bak and Bax to promote apoptosis [10]. In response to serum deprivation or cell density, SphK2 can also localize in the endoplasmic reticulum (ER) to produce S1P that can fuel a sphingolipid ‘salvage’ pathway, resulting in generated pro-apoptotic ceramide [34]. Two isoforms of SphK2 have been described [33, 42]. The best characterized short isoform (SphK2a or SphK2-S) is the one to which literature refers unless otherwise specified. The large isoform (SphK2b or SphK2-L) is not expressed in mice, and appears the predominant form in several human cell lines and tissues. SphK2b may be the more important in human physiologically [42].

In AD, a deregulation of the sphingolipid metabolism was originally shown in brain samples from AD patients and age-matched normal individuals, with reduced levels of S1P, together with elevated ceramide [26]. Cellular studies showed an alteration of the ceramide/S1P balance where Aβ treatment of neuronal [21] or glial [30] cells was associated with apoptosis caused by enhanced ceramide production or decreased protective S1P production. We further showed that a decrease of SphK1, S1P1 receptor expression together with an increased SPL expression in neurons were correlated to Aβ deposits in entorhinal cortex from human AD brains [8]. In line with these results, Couttas and coworkers showed that loss of activity of SphK1 and SphK2 was correlated with progression of AD lesions (Braak stages) [12]. The role of SphK2 particularly in AD remains controversial. While some authors showed a decrease of activity of SphK2 in temporal cortex and hippocampus [12], others reported an increase of SphK2 activity in the frontal cortex [49] of human AD brains.

Therefore, the involvement of SphK2 in AD processes needs further explorations. It could depend on deregulation of its expression or subcellular localization. To answer the question about SphK2 implication in the deleterious effects of Aβ in AD, we used brain tissues from patients with AD. We herein report for the first time that SphK2 expression is inversely associated with the density of amyloid deposits in frontal and hippocampal area of brains from AD patients and demonstrated a shift of SphK2 from the cytosol to the nucleus in AD neurons, accompanied by a marked increase of cleaved SphK2 in the AD brain.

## Materials and methods

### Validation of anti-SphK2 antibody

*E. coli* BL21 strains were transformed by one of the pJ414 constructs - PET21 vectors expressing either SphK1 (403AA) or SphK2 (674AA) with an HIS tag. Forty ml of an overnight pre-culture of transformants grown at 37 °C in DYT medium supplemented with kanamycin (100 μg/ml) was used to inoculate 200 ml of DYT-KAN media. After 2 h of growth at 37 °C, SphK proteins expression was induced by 5 mM of Isopropyl β-d-1-thiogalactopyranoside (IPTG). Aliquots of the cultures were made 5 h after induction. Protein expression was confirmed by western blot analysis with mouse anti-histidine (Cusabio, Ref. CSB-MA000011M0m). The specificity of the anti-SphK2CT rabbit polyclonal antibody (Sigma, Ref. SAB4502433) was confirmed by western blot.

### Human brain tissues

Paraffin embedded human brain tissues were provided by certified French biological resource centers from Lille (Neurobank Lille DC-2008-642) and Toulouse (Brain bank AC-2009-973) for immunohistochemistry and immunofluorescence studies. For western blot studies, human brain frozen tissues were provided by the national brain bank GIE Neuro-CEB (AC-2007-5) and the biological resource center of CHU Toulouse (Brain bank AC-2009-973). The utilization of postmortem material was approved by the corresponding biobank ethic committees. All cases were scored according to current criteria of NIA-Alzheimer’s Association [39]. The assessment included Braak and Thal neuropathology stages [5, 50].

### Immunohistochemistry

Post-mortem tissues from 25 AD patients were included in the immunohistochemical study. Characteristics of patients (age, gender, post mortem interval, Braak and Thal stages) are summarized in Table 1. Hemi-brains were fixed with formalin (4% in PBS) during approximately 1 month. Paraffin-embedded, formalin-fixed sections (4 μm) from frontal cortex and hippocampal area (entorhinal cortex and hippocampus) were deparaffinized in xylene and rehydrated in ethanol. Antigen retrieval was performed by immersing sections in boiling EDTA buffer (pH 9.0). Endogenous peroxidase and alkaline phosphatase were blocked by incubation of the sections for 5 min in Dual Endogenous Enzyme Block (Dako, Denmark). Sections were initially incubated with a primary antibody directed against β-amyloid (Dako, mouse clone 6 F/3D, Ref. M0872, 1:100) during 3 h at room temperature (RT). Sections were washed twice during 7 min in Tris-buffered NaCl solution with Tween 20 (pH 7.6, Dako). Immunostaining was revealed using BrightVision poly HRP-Anti-Mouse IgG (Immunologic, Netherlands) during 30 min at RT and treated with diaminobenzidine/hydrogen peroxide (DAB, Dako) for 5 min. After this first step, sections were washed for 10 min with Tris buffer saline (pH 7.6, Dako) before incubation with a primary rabbit polyclonal antibody directed against SphK2 (Sigma, Ref. SAB4502433, 1:50) overnight at 4 °C. The sections were washed twice during 7 min in Tris buffer saline (pH 7.6, Dako). Immunostaining was exposed using BrightVision poly AP-anti-Rabbit IgG (Immunologic) during 45 min at RT and treated with Liquid Fast Red (Abcam) for 30 min. Sections were counterstained with hematoxylin then mounted in Faramount Aqueous Mounting Medium (Dako). Once mounted, slides were scanned with a digital scanner NanoZoomer (Hamamatsu, Ref. [2].0-RS: C10730–13) to obtain high resolution virtual slides. Digitalized slides were analyzed with an imaging analysis system (ImageJ®). Morphometric investigations were carried out by a semi-automatic procedure on ImageJ. The number of neurons expressing SphK2, the percentage of Aβ stained surface and the number of amyloid deposits were quantified among the different cortical layers of the frontal and entorhinal cortex as well as in the CA1. Columns constituted of contiguous microscopic fields, from the pial surface to the white matter were drawn on each slide. As the fields were examined at a magnification of × 400, each field was 300 μM × 150 μM in size. As the thickness of the cortex appeared to be variable between the different sections, after the counting step, the columns were standardized to 10 fields [8, 18]. For cortical areas, field 1 corresponded to the cortex immediately under the pial surface and field 10 reached the white matter (Additional file 1). In each field, the number of neurons expressing a negative and a positive SphK2 stain were counted. Moreover, the number of Aβ focal deposits and the percentage of surface labeled by amyloid per field were determined and were reported on a database.

**Table 1:** Characteristics of patients included in the immunohistochemistry study

Variables	AD (*n* = 25)
Age at death (years)	
Median [IQR]	81.0 [73.0–87.0]
Range	59.0–96.0
Female gender, n (%)	18 (72)
Post mosterm interval	
Median [IQR]	21.0 [9.0–27.0]
Range	3.0–96.0
“A” Thal - n of patients (%)	
3 (4 and 5)	21 (84)
2 (3)	3 (12)
1 (1 and 2)	1 (4)
0 (None)	0 (0)
“B” Braak Stage - n of patients (%)	
3 (V and VI)	24 (96)
2 (III and IV)	1 (4)
1 (I and II)	0 (0)
0 (None)	0 (0)

### Preparation of human brain total homogenates

Fresh samples from frontal cortex and hippocampal area of 10 AD cases and 6 age-matched controls were used. No significant differences in sex and post-mortem interval were noted between AD patients and the control group. There was a difference in the age medians. As expected, AD patients had significantly higher Braak and Thal stages than the control group (Table 2). Samples of hippocampal area contained both entorhinal cortex and Ammon’s horn (including CA1). Moreover randomly selected samples of cerebellum from of AD (*n* = 5) and control (*n* = 3) were included as the cerebellar tissue exhibits a weak density of Aβ deposits even in the late phase of disease [50]. Frozen tissue samples were processed with Precellys® homogenizer and CK14 lysing kit (Bertin Instruments, Paris, France) containing lysis buffer (50 mM Tris-HCl pH 8.0, 5% SDS, 0.25 M saccharose, 200 mM NaCl and EDTA-free protease inhibitor cocktail). Samples were sonicated at 4 °C then centrifuged at 13000 g for 10 min.

**Table 2:** Characteristics of patients included in the western blot study

Variables	Controls (n = 6)	AD (n = 10)	*P*-value
Age at death (years)			
Median [IQR]	74.0 [70.5–80.0]	81.0 [78.0–85.0]	0.032
Range	67.0–86.0	62.0–89.0	
Female gender, n (%)	2 (33.3)	5 (55.5)	NS
Post mosterm interval			
Median [IQR]	17.5 [12.0–22.0]	8 [7.5–23.0]	NS
Range	4.0–36.0	6.0–50.0	
Missing (n)	1	0	
“A” Thal - n of patients (%)			< 0.001
3 (4 and 5)	0 (0)	8 (80)	
2 (3)	0 (0)	1 (10)	
1 (1 and 2)	1 (16.7)	1 (10)	
0 (None)	5 (83.3)	0 (0)	
“B” Braak Stage - n of patients (%)			< 0.001
3 (V and VI)	0 (0)	10 (100)	
2 (III and IV)	0 (0)	0 (0)	
1 (I and II)	5 (83.3)	0 (0)	
0 (None)	1 (16.7)	0 (0)	

### Preparation of nuclei-enriched fractions and cytoplasm-enriched fraction

The same cases except one control (AD = 10, Control = 5) were included in this experiment. Frozen tissue samples were lysed as aforementioned in HEPES buffer (pH 7.4) (20 mM HEPES, 10 mM KCl, 2 mM MgCl_2_, 1 mM EDTA, 0.25 M saccharose, and EDTA-free protease inhibitor cocktail). Nuclei-enriched fractions and cytoplasmic-enriched fractions were prepared by sequential centrifugation. Nuclei were pelleted by centrifugation (500 g for 10 min at 4 °C) then sonicated at 4 °C and centrifuged at 9000 g for 30 min, in Tris buffer (pH 7.4) (50 mM Tris-HCl, 250 mM NaCl, 1 mM EDTA, 0.1% Triton and EDTA-free protease inhibitor cocktail). Supernatants were removed, and the membrane and cytoplasmic fractions were separated by centrifugation at 16,000 g (30 min at 4 °C). Isolation of nuclear and cytosolic fractions was confirmed by western blot with primary antibodies directed against rabbit polyclonal PARP (GeneTex, Ref. GTX100573, 1:1000) and rabbit polyclonal LDH (GeneTex, Ref. GTX114525, 1:10,000).

### Western blot

Total protein concentration was assessed on the supernatant with the BCA Protein Assay (Interchim). Samples were heated at 95 °C for 5 min with 5% β-mercapto-ethanol, 0.05% bromophenol blue. 150 μg of total proteins were loaded by lane in precast gels with Stain-Free technology (4–15% Mini-PROTEAN® TGX Stain-Free™, Ref. 456–8083). The proteins were separated by electrophoresis at 90 V in a MiniProtean Tetra System (Bio-Rad Laboratories, Irvine, CA). After migration and semi-dry transfer (Transblot Turbo, Bio-Rad), nitrocellulose membranes were blocked with 5% skimmed milk, and washed 3 times with Tris-buffered saline buffer containing 0.05% Tween-20 (TBST). Blots were probed with a primary antibody directed against rabbit polyclonal SphK2 (Sigma, Ref. SAB4502433, 1:500) and β-amyloid (Sigma, Ref. A8354, 1:1000). After an overnight incubation at 4 °C, the membranes were washed with TBST, labeled with a peroxidase-conjugated anti-rabbit (dilution 1:3000) secondary antibody (Bio-Rad) and revealed by chemiluminescence (Clarity kit, Bio-Rad). Bands were quantified by densitometry with ImageLab (Bio-Rad) software. The density of total protein, detected by UV light, was used to normalize the signals [20, 23].

### Immunofluorescence

Post-mortem tissues from 9 AD and 6 controls were included in the immunofluorescence study (Table 3). There were no significant differences in age, sex and post mortem interval between the groups used for the immunofluorescence study. Most of the AD subjects were staged Braak V-VI and Thal 4 to 5. Control subjects included cases with respectively low Braak (I-II) or Thal (1–2) stages. Immunofluorescent staining of SphK2 and MAP2 was performed on paraffin-embedded, formalin-fixed human brain sections. The human brain sections were deparaffinized in xylene and rehydrated in ethanol. Antigen retrieval was performed by immersing sections in boiling EDTA buffer (pH 9.0). Human brain sections were washed in PBS, permeabilized with PBS-Triton 0.1% for 10 min. To avoid the autofluorescence, sections were incubated in True black 1X (Ozyme) for 1 min. Nonspecific antibody reactions were blocked by incubation in solution containing 5% BSA for 1 h. Sections were incubated with mouse monoclonal primary anti-MAP2 (Life technologies, Ref. MA512826, 1:100) and anti-SphK2 (Sigma, Ref. SAB4502433, 1:50) antibodies. Secondary antibodies included goat anti-rabbit IgG (Life technologies, Alexa Fluor® 543 conjugate, Ref. A-11010, 1:1000) and labeled goat anti-mouse IgG (Life technologies, Alexa Fluor® 488, Ref. A-11001, 1:1000). DAPI was used as a nuclear counterstain (final concentration of 1 μg/mL). It also allowed to visualize the amyloid deposits (Additional file 2)*.* Human brain sections were mounted with Fluorescence mounting medium (DAKO S3023). Confocal laser microscopy was performed on a Zeiss, LSM 780 apparatus. Several fields of view (> 300 cells) were analyzed for each group. The confocal composite image (merge) was analyzed using ImageJ 1.51o software. Morphometric investigations were carried out to quantify the percentage of surface stained by SphK2 antibody. SphK2 staining in neurons was estimated on the total surface of each neuron, using a staining threshold taking into account the background. The percentage of SphK2 stained surface was further analyzed in two cell compartments leading to a ratio of nuclear versus cytoplasmic stained surfaces. The same nuclear/cytoplasmic ratio was applied for analysis of intensity of SphK2 staining. To overcome experimental biases, the percentage of colocalization between MAP2 and SphK2 and the percentage of colocalization between DAPI and SphK2 were determined.

**Table 3:** Characteristics of patients included in the immunofluorescence study

Variables	Controls (n = 6)	AD (n = 9)	*P*-value
Age at death (years)			
Median [IQR]	70.0 [68.0–73.0]	81.0 [72.0–85.0]	NS
Range	58.0–82.0	69.0–88.0	
Female gender, n (%)	2 (33.3)	5 (50.0)	NS
Post mosterm interval			
Median [IQR]	10.0 [5.5–23.0]	24.5 [16.0–29.0]	NS
Range	5.0–24.0	8.0–44.0	
“A” Thal - n of patients (%)			< 0.001
3 (4 and 5)	0 (0)	8 (88.9)	
2 (3)	0 (0)	1 (11.1)	
1 (1 and 2)	4 (66.7)	0 (0)	
0 (None)	2 (33.3)	0 (0)	
“B” Braak Stage - n of patients (%)			< 0.001
3 (V and VI)	0 (0)	9 (100)	
2 (III and IV)	1 (16.7)	0 (0)	
1 (I and II)	3 (50)	0 (0)	
0 (None)	2 (33.3)	0 (0)	

### Data analysis

Statistical analysis was carried out with a multilevel linear mixed model to take into account non independent data. For cortical area, due to the poor representativeness of fields 1 (non tissular zone and pial surface) and 10 (proximal white matter), these fields were not included in statistical analysis. To avoid experimental bias related to the number of neurons per field, the immunohistochemistry data were expressed as percentage of variation relative to the total number neurons. For correlation analyses, the Spearman’s correlation coefficient, R, was determined. Correlations were estimated as significant at *p* <  0.05. Comparisons between groups were analyzed using one-way analyses of variance (ANOVAs). Statistical analyses were performed using the Statview 5.0 software.

## Results

### SphK2 antibody is specific for SphK2 and does not cross-react with the SphK1 isoenzyme

**Fig. 1:**
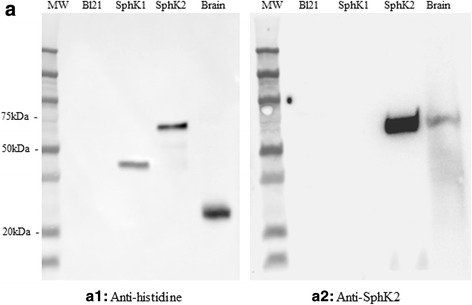
Validation of anti-SphK2 antibody. *Escherichia coli* strain BL21 overexpressing His-tagged full length SphK1, His-tagged full length SphK2, and human brain lysate were analyzed by Western blotting using anti-Histidine (a1) and anti-SphK2 antibodies (a2)

We used a rabbit polyclonal SphK2 antibody raised against amino acid 580–629 sequence. The western blots carried out on SphK1 and SphK2 recombinant proteins and on human brain lysate, confirmed the specificity of the antibody for SphK2 recombinant protein with no cross-reaction for the SphK1 isoenzyme (Fig. 1a2). No other bands were observed with SphK2 antibody in whole control brain lysate.

### Immunohistochemical studies

**Fig. 2:**
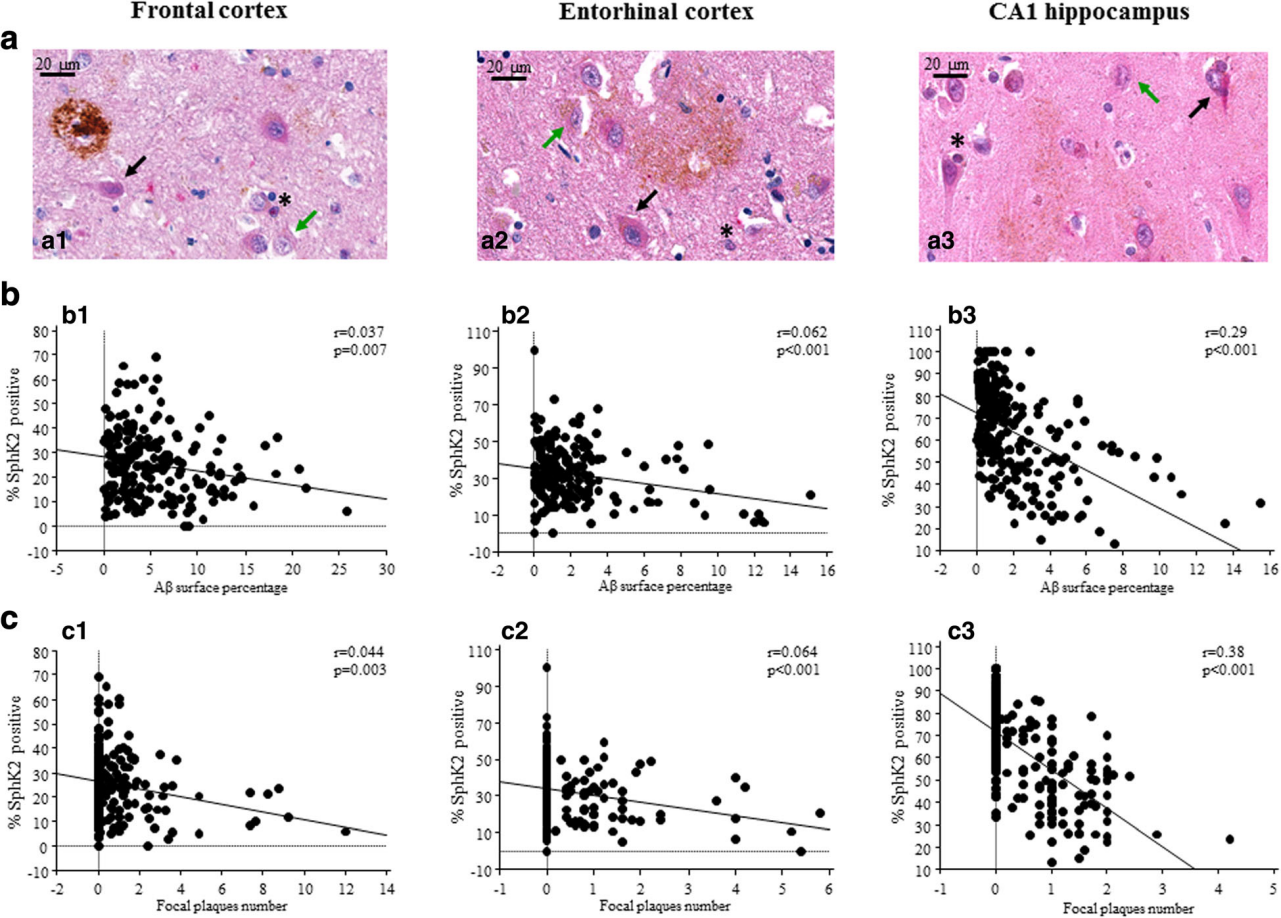
Immunohistochemical study of SphK2 protein expression and Aβ deposits. **a** Double labeling of SphK2 and Aβ in AD brain sections (*n* = 25) from frontal (a1) entorhinal cortex (a2) and CA1 hippocampus (a3). Images were taken at 40× magnification (Scale bars represent 20 μm). Black arrows show SphK2 positive neurons, green arrows show SphK2 negative neurons and asterisks show SphK2 positive oligodendrocytes. **b** Scatter plots depicting the relationship between density of amyloid deposits or Aβ surface percentage (x axis) and SphK2 expression (y axis) in frontal cortex (b1), entorhinal cortex (b2) and CA1 (b3). Regression lines are displayed. **c** Scatter plots depicting the relationship between number of focal plaques number (x axis) and SphK2 expression (y axis) in frontal cortex (c1), entorhinal cortex (c2) and CA1 (c3). Regression lines are displayed. The results are based on evaluation of 5305 neurons in the frontal cortex, 3238 neurons in entorhinal cortex and 3198 neurons in CA1

Most of the subjects presented a high number of neurofibrillary tangles as well as of amyloid deposits (Braak V-VI and Thal 4 to 5; Table 1). In both studied cortical areas, thickness variability was noticed and could be related to atrophy, which is a common feature in AD. The Aβ deposits were more frequent in cortical layers II and III, mostly represented in fields 2 to 6 (Additional file 3). There was no correlation between the density of neurons and Aβ deposits (in three locations, *p* > 0.5 for all regressions), whether Aβ deposits were evaluated as the number of focal deposits or the percentage of amyloid areas (p > 0.5 for all regressions). SphK2 staining was mainly observed in neurons (Fig. 2a), and likely in the nucleus of cells exhibiting the hallmarks (size, shape, specific location) of oligodendrocytes. SphK2 staining was also observed less frequently in astrocytes. In neurons, the immunostaining of SphK2 was both nuclear and cytoplasmic. Because the nuclear immunostaining of SphK2 was difficult to quantify, our analysis focused on cytoplasmic SphK2 expression to avoid experimental bias. The cytoplasmic SphK2 expression was heterogeneous between the three analyzed structures (F_(2,647)_ = 350.8, *p* <  0.0001). The percentage of SphK2 positive neurons was higher in CA1 (vs frontal or entorhinal cortex, p <  0.0001 for both comparisons) and lower in frontal vs entorhinal cortex (p <  0.0001).

### Negative correlation between amyloid deposits and the percentage of SphK2 positive neurons in AD brain

The percentage of SphK2 positive neurons (cytoplasm) was inversely correlated with the amount of amyloid deposits (Aβ surface percentage) in the frontal cortex (*r* = 0.037, *p* = 0.007; Fig. 2b1), in the entorhinal cortex (*r* = 0.062, *p* <  0.001; Fig. 2b2) and in CA1 (*r* = 0.29, p <  0.001; Fig. 2b3). This negative correlation was also found for the focal plaques (frontal cortex: *r* = 0.044, *p* = 0.003; entorhinal cortex: *r* = 0.064, p <  0.001; and CA1: *r* = 0.38, p <  0.001; Fig. 2c). From the coefficient values, we noted that the relationship between the amount of amyloid and the percentage of SphK2 positive neurons was stronger in the CA1 area than in the cortical regions (frontal and entorhinal).

### Frontal cortex and hippocampal AD brain extracts show high amyloid content

**Fig. 3:**
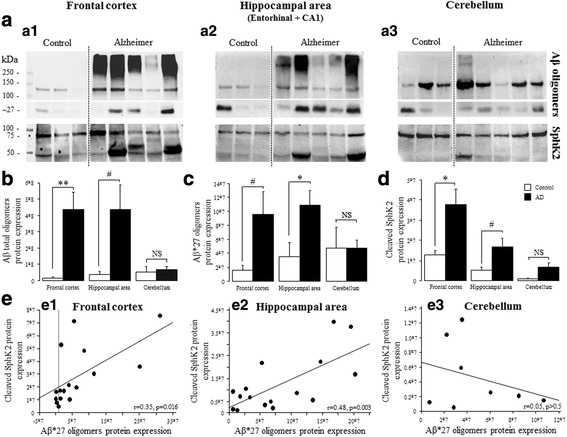
Correlation between the proportion of cleaved SphK2 and Aβ*27 oligomers in frontal cortex, hippocampal area and cerebellum. **a** Representative blot showing Aβ oligomers (upper panel) and SphK2 (lower panel) full length and cleaved proteins in frontal cortex (a1), hippocampal area (entorhinal cortex and CA1) (a2) and cerebellum (a3) tissue samples. Aβ oligomers and SphK2 expression were normalized using stain-free imaging technology. The relative abundance of total Aβ oligomers (**b**) and Aβ*27 oligomers (**c**) was analyzed in the three structures. **d** Cleaved SphK2 expression, in control (*n* = 6) and AD (*n* = 10) groups in frontal cortex, hippocampal area and cerebellum (control *n* = 3 and AD *n* = 5). Statistical significance was determined using one-way ANOVA. Columns, mean; bars, SEM. *p < 0.05, **p < 0.01, # *p* < 0.08 vs control group. **e** Scatter plots depicting the relationship between Aβ*27 oligomers (x axis) and cleaved SphK2 expression (y axis) in frontal cortex (e1), hippocampal area (e2) and cerebellum (c3). Regression lines are displayed

The amount of Aβ was assessed in human brain lysates from the frontal cortex, hippocampal area (entorhinal cortex and CA1) in AD and control groups. To better appreciate the relation between SphK2 and Aβ deposits, we analyzed samples of cerebellum, a brain area where Aβ deposits are scarce and appear late in the evolution of the disease [50] (Fig. 3a, *top row*). Western blot studies revealed the presence of the β-amyloid precursor protein (APP, approximately 130 kDa band) along with a set of apparent supramolecular Aβ assemblies. High-molecular weight Aβ oligomers or multiples of trimeric Aβ oligomers have been previously characterized in human AD brain tissue [32, 51]. Quantitative analyses did not demonstrate significant differences between control and AD groups for APP (*p* > 0.5).

In addition to 150–250 kDa bands corresponding to higher MW Aβ oligomers, bands of 27 kDa were detected. This molecular masses apparently correspond to multiples of trimeric Aβ and could thus represent hexameric (Aβ*27) Aβ assemblies. High levels of total Aβ (high MW oligomers and Aβ*27) were observed in AD group in comparison with the control group (F_(1,38)_ = 10.7; *p* = 0.002). When each structure was considered separately (Fig. 3b), statistical analysis revealed a significant increase of total Aβ in frontal cortex (*p* = 0.009) and an increase at the margin of statistical significance in the hippocampal area (*p* = 0.06). No difference was found in the cerebellum (p > 0.5).

With regard to the Aβ*27 oligomers, there was a significant difference between groups (F_(1,38)_ = 7.9; *p* = 0.008), with a significant increase in hippocampal area (*p* = 0.028; Fig. 3c) and an increase in the frontal to the margin of the significance (*p* = 0.08). No difference was found in cerebellum (*p* = 0.99).

Higher MW Aβ oligomers (F_(1,38)_ = 8.6; *p* = 0.006) content were markedly increased in frontal cortex (p = 0.02) but not significantly in hippocampal area (*p* = 0.095) and in the cerebellum (*p* = 0.4), in AD group (data not shown).

These data are in accordance with the evolution of Aβ deposits and the distinct sequences in which the regions of the brain are hierarchically involved according to the Thal stages (Phase 1: neocortical Aβ deposits, Phase 2: hippocampal area deposits, Phase 5: cerebellum is the latest structure affected by Aβ deposits) [50].

### Specific increased expression of a cleaved SphK2 form in AD brains

The SphK2 immunoblots showed two major bands in brain lysates, a 75 kDa band corresponding to the large isoform of SphK2 (SphK2b) and a protein of lower molecular weight which was considered as a cleaved fragment of SphK2 (Fig. 3a, *low row*). A cleaved fragment of SphK2 has been previously described in extraneural cell models (Jurkat T cells, murine fibroblasts, spleen cells) in relation to apoptotic process [53].

The quantification of total SphK2 (full length and cleaved forms) revealed an increase of its expression in AD samples, in frontal cortex (F_(1,14)_ = 5.4; *p* = 0.04) but not in the hippocampal area and cerebellum (*p* > 0.5 for both comparisons). However, full length SphK2 was not different between AD and control groups (data not shown) (*p* > 0.05). In contrast, the cleaved SphK2 fragments (Fig. 3d) was higher in AD extracts as compared to control (F_(1,38)_ = 7.6; *p* = 0.009). More specifically, an increase was observed in the frontal cortex (F_(1,14)_ = 5.8; *p* = 0.03) and in the hippocampal area, to the margin of the significance (F_(1,14)_ = 4.1; *p* = 0.06). No difference was found in cerebellum (*p* > 0.08).

Correlation analyses were made to confirm relationships between individual expression of a cleaved SphK2 form and Aβ levels (Fig. 3e). The individual expression of cleaved SphK2 form correlated positively with Aβ*27 levels in frontal cortex (*r* = 0.35, *p* = 0.016; Fig. 3e1) and in hippocampal area (*r* = 0.48, *p* = 0.003; Fig. 3e2), but not in cerebellum (p > 0.5; Fig. 3e3).

A positive correlation was also noted between higher MW Aβ oligomers and cleaved SphK2 in frontal cortex (*r* = 0.47, p = 0.003) and in hippocampal area (*r* = 0.73, *p* = 0.001) (data not shown). In the cerebellum, higher MW Aβ oligomers were expressed at a low level in one AD case (Fig. 3a3). Cleaved SphK2 was also found in this case.

### The subcellular localization of SphK2 is altered in AD brains

**Fig. 4:**
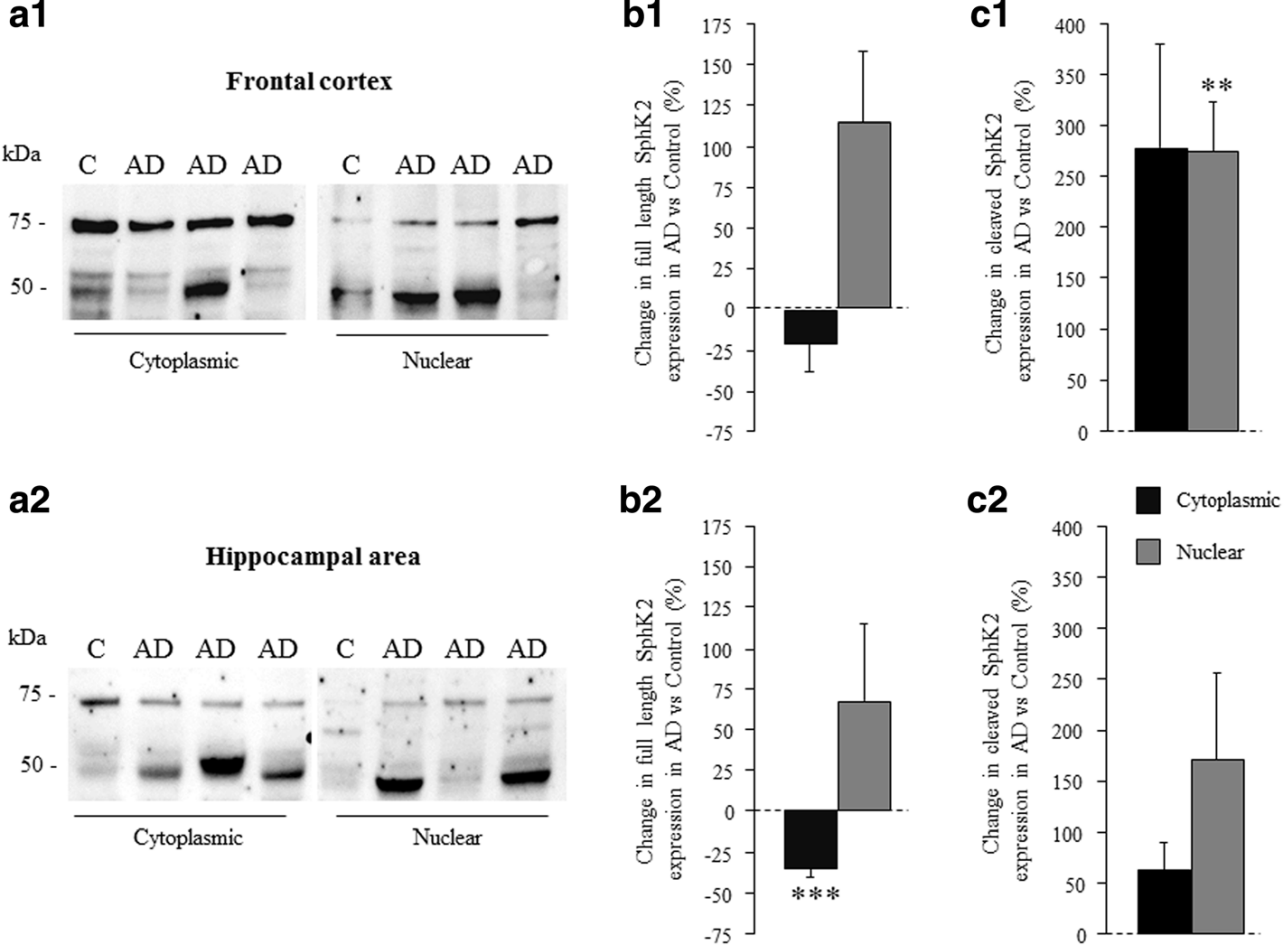
SphK2 protein expression is increased in nuclear compartments in AD group. Subcellular localization of both full length and cleaved SphK2 was analyzed by western blot. **a** Representative western blot showing SphK2 expression in cytoplasmic and in nuclear fraction of control (n = 5) or AD groups (n = 10) of tissue samples from the frontal cortex (a1) or the hippocampal area (a2). Relative expression of full length SphK2 (**b**) and cleaved SphK2 (**c**) proteins in cytoplasmic or nuclear fractions was analyzed in the frontal cortex (b1-c1) and the hippocampal area (b2-c2) of tissue extracts in AD group as compared to control group. After normalization (using stain-free imaging technology), data were expressed as percentage of SphK2 expression levels in AD versus control group. Statistical significance was determined using one-way ANOVA test. Columns, mean; bars, SEM. **p < 0.01, ***p < 0.001 vs control group

To analyze the subcellular localization of both full length and cleaved SphK2, cytoplasmic- and nuclei-enriched fractions were prepared from frontal cortex and hippocampal area. In line with data presented in Fig. 3, the cleaved SphK2 expression was markedly increased in AD samples when compared to controls (F_(1,58)_ = 5.7; *p* = 0.02; Fig. 4a) (data not shown).

The quantification of total SphK2 (full length and cleaved forms) revealed a differential expression between the two subcellular fractions in control group (F_(1,18)_ = 12.9; *p* = 0.002). SphK2 was less expressed in the nuclei-enriched fraction (1.22^E^8 ± 0.23^E^8 densitometry units) than in the cytoplasmic-enriched fraction (2.47^E^8 ± 0.25^E^8 densitometry units). This difference was found in the frontal cortex (*p* = 0.04) and in the hippocampal area (*p* = 0.01). In AD group (F_(1,38)_ = 0.35; *p* = 0.5), no difference was found between the nuclear (2.75^E^8 ± 0.63^E^8 densitometry units) and the cytoplasmic (3.22^E^8 ± 0.48^E^8 densitometry units) (data not shown).

For each subcellular fraction, we analyzed separately full length and cleaved SphK2 fragments in AD group as compared to control. The results are expressed as percentage variation in AD versus control group (Fig. 4b and c).

Full length SphK2 expression was increased in the nuclei enriched fraction (F_(1,28)_ = 3.9; *p* = 0.050) but was decreased in cytoplasmic enriched fraction (F_(1,28)_ = 5.4; *p* = 0.027) in AD group relative to the control (Fig. 4b). Post-hoc analyses showed a reduction of cytoplasmic full length SphK2 mainly in the hippocampal area (*p* < 0.001).

A rise of cleaved SphK2 (Fig. 4c) was observed in nuclear fractions (F_(1,28)_ = 10.4; *p* = 0.003) and in cytoplasmic fractions (F_(1,28)_ = 3.9; p = 0.050) in AD group relative to control group especially in frontal cortex (nuclear *p* = 0.0015 et cytoplasmic *p* = 0.08; Fig. 4c1). To a lesser extent, yet not significantly, this change of expression was seen in hippocampal area (nuclear *p* = 0.17 and cytoplasmic *p* = 0.13; Fig. 4c2).

These data suggest a shift of full length SphK2 from cytosol to the nucleus examined in all brain structures, accompanied by an accumulation of the cleaved SphK2 in the nucleus that would be more significant in the frontal cortex, in AD.

### Immunofluorescence study shows that SphK2 is preferentially localized in the nucleus

The density of neurons and of amyloid deposits were inversely correlated in the frontal (*r* = 0.14, *p* = 0.0004) and entorhinal cortex (*r* = 0.15, *p* = 0.002), but not in the CA1 (*r* = 0.02, *p* = 0.25). This negative correlation was only related to the presence of focal deposits in all structure (frontal cortex: *r* = 0.13, *p* = 0.0006, entorhinal cortex: r = 0.13, *p* = 0.0045, CA1: *r* = 0.11, *p* = 0.005) (data not shown).

**Fig. 5:**
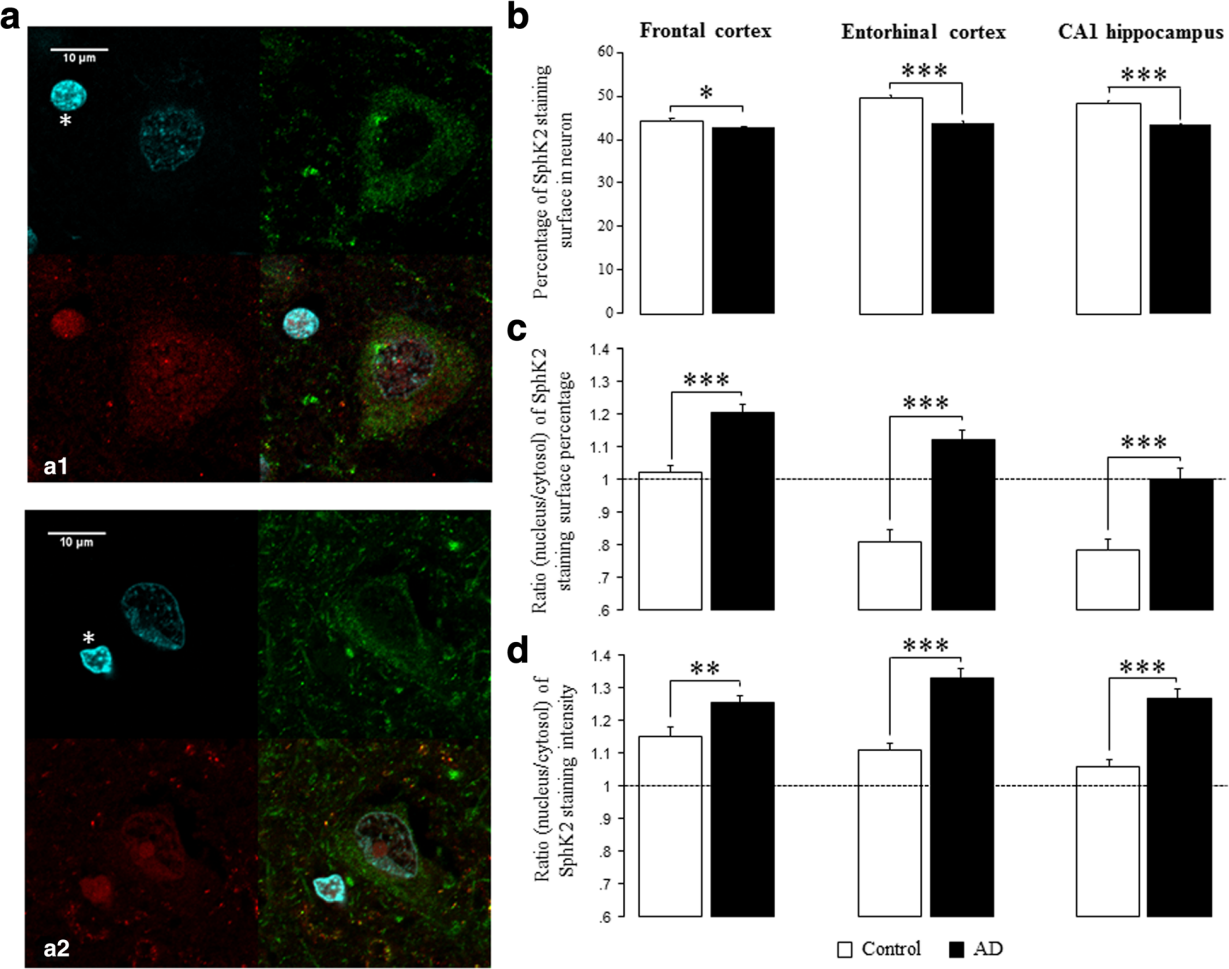
Disruption of SphK2 localization within neurons in AD brain. **a** Representative confocal micrographs of SphK2 subcellular localization in fixed human frontal cortex tissue, in control (a1; *n* = 6) and AD (a2; *n* = 9) groups. Images were taken at 63× magnification (Scale bars indicate 10 μm). A TRITC-conjugated secondary antibody was used for SphK2 (red) and a FITC-conjugated secondary antibody was used for MAP2 (green). Nuclei were stained with DAPI (cyan). The confocal composite image (merge) was analyzed using ImageJ 1.51o software. The results are based on the evaluation of 493 neurons in AD group (frontal cortex: 187; entorhinal cortex: 150; CA1: 156) and 346 neurons in control group (frontal cortex: 133; entorhinal cortex: 90; CA1: 123). SphK2 staining was mainly observed in neurons and oligodendrocytes (asterisk). In neurons, the immunostaining of SphK2 was both nuclear and cytoplasmic. **b** Percentage of SphK2 staining surface was quantified in neurons. **c** SphK2 staining ratio between nuclear and cytoplasmic surface (%) in control and AD neurons for the three areas. **d** SphK2 staining intensity ratio (nuclear/cytoplasmic) in control and AD neurons for the three areas. Columns, mean; bars, SEM *p < 0.05, **p < 0.01, ***p < 0.001 vs control

We first examined the distribution of SphK2 in the frontal cortex, entorhinal cortex and CA1 then its subcellular localization. With SphK2 immunostaining both full length and cleaved fragments were stained. The immunostaining of SphK2 was mainly seen in neurons and oligodendrocytes (Fig. 5a). In neurons, SphK2 was essentially seen in the nucleus in AD contrary to the control group in which SphK2 was mostly expressed in the cytosol (intensity: *p* < 0.001; neuron area (%): p < 0.001 for both comparisons). At regional level, SphK2 was not homogeneous (intensity: F_(2,704)_ = 8.9; *p* = 0.05; neuron area (%): F_(2,704)_ = 5.5; p = 0.005). Indeed, SphK2 was more expressed in the CA1 (intensity: *p* = 0.026 vs frontal cortex, p = NS vs entorhinal; neuron area (%): p = 0.05 vs frontal cortex, *p* = 0.001 vs entorhinal) (data not shown) as compared to both cortical regions.

In agreement with data shown in Fig. 2, immunofluoresence microscopy revealed that the percentage of SphK2 stained surface in neuron (nuclear and cytoplasmic compartments) was decreased (F_(1,837)_ = 49.0; *p* < 0.0001; Fig. 5b) in the AD group as compared to control for all structures (frontal cortex: *p* = 0.033; entorhinal cortex and CA1: p < 0.0001). The nuclear/cytoplasmic ratio of SphK2 stained surface was increased in AD group (F_(1,837)_ = 113.5; p < 0.0001). This change of expression was seen in the frontal cortex, the entorhinal cortex and the CA1 (p < 0.001 for all comparisons) as compared to the control subjects, demonstrating a disturbance of the SphK2 subcellular localization (Fig. 5c). This observation was reinforced by the increase of SphK2 nuclear/cytoplasmic ratio of staining intensity in AD group, for the three structures (*p* ≤ 0.001 for the three structures) (Fig. 5d).

## Discussion

A deregulation of sphingolipid metabolism and more particularly S1P signaling has been recently stressed in AD (reviewed in [7, 9, 28, 38, 52]). Initial work in neuronal cell models showed that apoptosis induced by Aβ peptide was dependent on increased production of pro-apoptotic ceramide [30] or decreased activity of SphK1 leading to decreased content of pro-survival S1P [21]. This deregulation of the ceramide/S1P balance with increase in ceramide and decrease in S1P was seen in patients to correlate with the level of Aβ peptide and phosphorylated tau [26]. More recently, two independent studies established an alteration of S1P metabolism with a decrease in SphK1 or SphK2 expression and enzymatic activity and increase in S1P lyase, the enzyme that degrades S1P [8, 12]. Again, enzymatic deregulation leading to decreased S1P correlated with Aβ deposits and progression of AD lesions [8, 12].

Besides the likely role for SphK1 isoform, SphK2 deregulation may contribute more significantly to AD lesions and progression. Indeed, SphK2 was found as the predominant isoenzyme in the rodent brain [3, 31, 33], and SphK2 activity and mRNA levels are considerably higher than that of SphK1 [3, 12]. Using mice with germline knockout of SphK2, it was recently shown that SphK2 is responsible to the largest part for S1P synthesis in the brain [31]. As aforementioned, the role of SphK2 has been examined in AD patients yet the results remain so far controversial. If a decrease in S1P level was linked to the reduction of SphK2 activity in the hippocampus and the temporal cortex [12], an increase of SphK2 activity has been reported in the frontal cortex [49]. These ambiguous results may simply reflect the complexity of SphK2 regulation and function. Depending on its subcellular compartment, SphK2 can generate S1P that will favor cell death or proliferation and survival [40].

Until now, the lack of commercial SphK2 antibodies validated for western blotting and immunohistochemistry has remained a drawback for a better understanding of the specific functions of SphK2. In this study, we used a SphK2 antibody raised against the C-terminal sequence of SphK2, the specificity of which for the SphK2 isoenzyme was confirmed using human SphK1 and SphK2 recombinant proteins.

We herein report that a decrease in cytoplasmic SphK2 protein expression correlates with the density of amyloid deposits in AD brains. Interestingly, we observed that the equilibrium between cytoplasmic and nuclear SphK2 was disrupted and showed that SphK2 is preferentially localized in the nucleus in AD brain extracts as compared to control extracts, with a marked increase of cleaved SphK2.

More precisely, we showed that cytoplasmic expression of SphK2 in neurons was inversely correlated to Aβ deposits, whatever their type (diffuse or focal) in the CA1 and to a lesser extent in the frontal and entorhinal cortex. This is in keeping with our previous results on SphK1 cytoplasmic expression, which was also negatively correlated to the density of Aβ deposit [8], suggesting the involvement of both isoenzymes in the deregulation of this signaling pathway. Western blot analyses corroborated the absence of variation of SphK2 expression in whole brain lysates of AD patients when compared to control brains as previously reported [8, 49]. Cieslik et al., using PC12 cells, similarly showed that exposure to Aβ_1–42_ had no effect on SphK2 [11]. These results showing an absence of variation of total SphK2 at both cellular and tissular levels suggest there could be a change in subcellular localization of SphK2 without any alteration of its total expression. As a matter of fact, both nuclei-enriched and the cytoplasmic-enriched fractions showed an alteration of SphK2 expression with a loss of expression in cytoplasm accompanied with an increased expression in nucleus in hippocampal area and to a lesser extent in frontal cortex. Immunofluorescence studies confirmed that nuclear and cytoplasmic expressions of SphK2 were modified in favor of a cytoplasmic decrease and a nuclear increase expression in AD brains.

Taking advantage of an antibody directed against the C-terminal portion of SphK2, the presence of a low molecular weight protein (~ 50 kDa) could be detected on immunoblots. This form matches with the truncated fragment of SphK2 cleaved by caspase-1 at the level of the D138 according to Weigert et al., and online software ExPASy (PeptideCutter) [53]. In this truncated fragment, all the domains of SphK2 are preserved including the catalytic domain and the ligand binding domain, suggesting that cleaved SphK2 could preserve its enzymatic activity. Its distribution in both compartments is in agreement with theoretical predictions based on online software WoLF PSORT 20, which establishes that cleaved SphK2 could be localized in both cytoplasmic and nuclear compartments. Caspase-1-mediated cleavage of SphK2, by removing the nuclear localization signal (NLS), would prevent the entry in the nucleus of a cytosolic cleaved SphK2. This is in favor of a cleavage of SphK2 in the nucleus. Actually, it has been reported that inactivated caspase-1 can be translocated to the nucleus and processed into an active form in parallel with apoptosis [35]. Our data show that an increase of cleaved SphK2 in AD group correlated positively with the amount of amyloid oligomers, particularly in hippocampus and in entorhinal cortex. Yet, the significance and function of such cleaved SphK2 requires further interrogation.

Similar to SphK1, SphK2 has a pro-survival effect when localized in the cytoplasm from where it can be targeted to the plasma membrane to produce S1P, which can be then secreted to exert autocrine and paracrine effect through its specific receptors [40]. Thus, our results suggest that, during AD, there is a decrease in the pro-survival cytoplasmic SphK2 similar to previous findings showing a decrease in pro-survival cytoplasmic SphK1 [8, 12]. We also previously reported that S1P receptor subtype 1 was decreased in AD [8] and recent data suggest that pharmacological agonists of S1P1 (FTY720, SEW2871) can improve cognitive function in AD rat model [1, 2]. Collectively, these data indicate that cytosolic SphK1/SphK2 signaling and potential subsequent S1P receptor signaling could be linked to a pro-survival role of S1P in AD pathogenesis, and that strategies aimed at rescuing impaired cytosolic SphK1/SphK2 signaling could be beneficial for patients.

The nuclear SphK2 signaling appears much more complex. It could be deleterious in AD pathogenesis. Interestingly, in cancer models, retinoic acid signaling was found to be deregulated by nuclear SphK2/S1P signaling, with S1P acting as an antagonist for the retinoic acid receptor (RAR) beta [47, 48]. Retinoic acid represents an antioxidant that can affect cognition [4, 43]. More specifically, retinoic acid can modulate the hippocampus-dependent memory influencing the neuronal plasticity (changes in synaptic strength, numbers of synapses and neurons) [36]. The disruption of retinoid signaling has been implicated in the pathogenesis of AD [22]. The expressions of genes that are engaged in the production of Aβ including β-secretase enzyme (BACE), presenilin 1 or presenilin 2 are regulated by retinoids [29, 41]. Finally, the protective role of retinoic acid in the pathogenesis of AD has been demonstrated in transgenic APP/PS1 mouse model. Indeed, its prolonged administration produced a reduction of amyloid deposits and lead to an improvement of memory [15]. Thus, the disruption of acid retinoic signaling by the nuclear SphK2/S1P signaling may have an implication of neurodegenerative processes in AD and in the memory disorders. On the other hand, nuclear SphK2 may have a beneficial effect via its role in epigenetic regulation, on synaptic plasticity and memory processes. Indeed, the nuclear S1P produced by SphK2 inhibits histone deacetylases HDAC1/2 and regulated histone acetylation [24]. FTY720, a synthetic analog of sphingosine which is phosphorylated by nuclear SphK2, induced an improvement of memory process [19, 25] and a better survival of neuronal progenitors in hippocampus [19]. It could be hypothesized that the nuclear-localized SphK2 is involved in a compensatory attempt process to restore neuronal function.

## Conclusions

The cellular functions of SphK2 are complex and the mechanism(s) that may drive the differences in its subcellular localization remain(s) largely unanswered question and will require further investigation. Similarly, future work is needed to understand the significance of the presence of cleaved SphK2 in the neuron nuclei. Nevertheless, from our findings it is reasonable to suggest that a change in the subcellular localization of the S1P generating SphK2 may compromise the well-established pro-survival cytosolic S1P by favoring the production of nuclear S1P associated with deleterious effects in AD pathogenesis.

## Additional files

Additional file 1: Virtual slides and counting method. (a) Virtual slide obtained from hippocampal area section. The section is double labeled for SphK2 and Aβ. (b) Representative scale matrix designed on entorhinal cortex used for neurons and Aβ deposits counting. Boxes extend from pial surface to white matter. The number of fields mainly depends on the thickness of the cortex. To compare results from different individuals, it was necessary to standardize the number of fields. Thus, after the counting step, the columns were standardized to 10 fields [18]. (TIFF 319 kb)

Additional file 2: Visualization of amyloid deposits was carried out using a DAPI staining. Our preliminary studies showed that DAPI stains the nuclei as well as plaques in gray matter. In order to validate that DAPI stained extracellular deposits are amyloid deposits, an immunofluorescent staining of Aβ peptide was realized with a mouse primary antibody (Dako, mouse clone 6 F/3D, Ref. M0872, 1:100). Immunofluorescence study was performed on paraffin-embedded, formalin-fixed human brain sections. Secondary antibody of goat anti-mouse IgG (Life technologies, Alexa Fluor® 488, Ref. A-11001, 1:1000) was used for visualization. DAPI was used as a nuclear counterstain (final concentration of 1 μg/mL). The merge confocal composite image was analyzed with ImageJ 1.51o software and was confirmed the colocalization. (TIFF 284 kb)

Additional file 3: Percentage of amyloid area according to fields. This percentage was calculated on the whole population of 25 cases. Field 1 corresponded to the cortex immediately under the pial surface and field 10 reached the white matter. Due to the poor representativeness of fields 1 (non tissular zone and pial surface) and 10 (proximal white matter), they were not included in statistical analysis for the cortical areas. The distribution of cortical layers was consistent with previously reported morphological studies ([18]; [17]). For instance, in frontal and entorhinal cortices, the cortical layer I was principally found in fields 1 and 2, cortical layers II and III were mostly represented in fields 2 to 6, layer IV was confined in fields 6 to 8, and layers V and VI were found in fields 7 to 10. Moreover, the Aβ deposits were more frequent in cortical layers II and III. As the fields were examined at a magnification of × 400, each field was 300 μM × 150 μM in size. (TIFF 35 kb)
