# Supplementary figures and images for: Neuronal sphingosine kinase 2 subcellular localization is altered in Alzheimer’s disease brain
Source: Acta Neuropathol Commun. 2018 Apr 3;6:25. doi: 10.1186/s40478-018-0527-z (PMC5883421; doi:10.1186/s40478-018-0527-z)

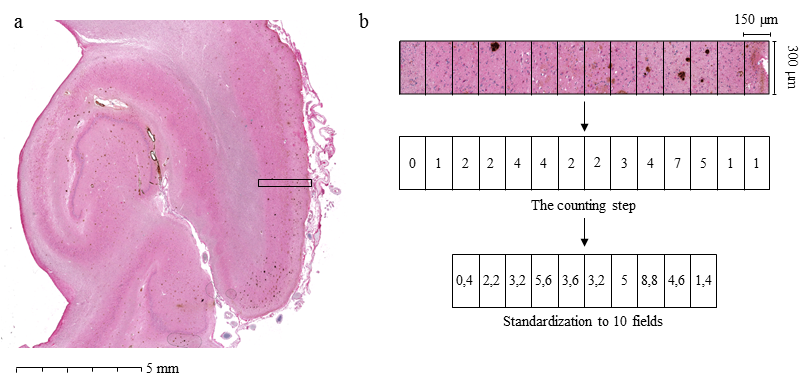

Supplement: Supplementary file 1 — Virtual slides and counting method. (a) Virtual slide obtained from hippocampal area section. The section is double labeled for SphK2 and Aβ. (b) Representative scale matrix designed on entorhinal cortex used for neurons and Aβ deposits counting. Boxes extend from pial surface to white matter. The number of fields mainly depends on the thickness of the cortex. To compare results from different individuals, it was necessary to standardize the number of fields. Thus, after the counting step, the columns were standardized to 10 fields [18]. (TIFF 319 kb) [file 40478_2018_527_MOESM1_ESM.tif]

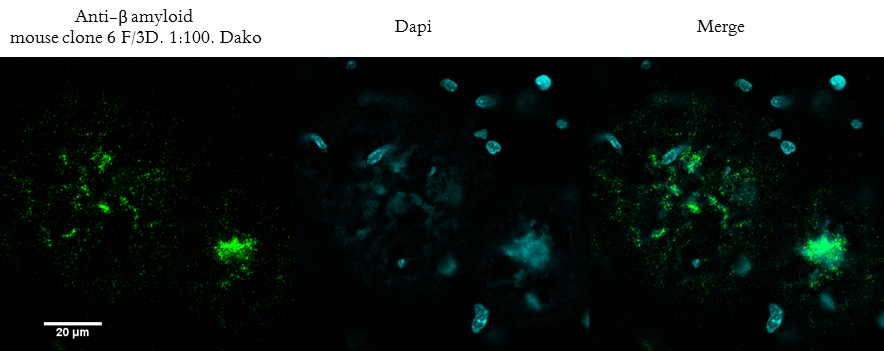

Supplement: Supplementary file 2 — Visualization of amyloid deposits was carried out using a DAPI staining. Our preliminary studies showed that DAPI stains the nuclei as well as plaques in gray matter. In order to validate that DAPI stained extracellular deposits are amyloid deposits, an immunofluorescent staining of Aβ peptide was realized with a mouse primary antibody (Dako, mouse clone 6 F/3D, Ref. M0872, 1:100). Immunofluorescence study was performed on paraffin-embedded, formalin-fixed human brain sections. Secondary antibody of goat anti-mouse IgG (Life technologies, Alexa Fluor® 488, Ref. A-11001, 1:1000) was used for visualization. DAPI was used as a nuclear counterstain (final concentration of 1 μg/mL). The merge confocal composite image was analyzed with ImageJ 1.51o software and was confirmed the colocalization. (TIFF 284 kb) [file 40478_2018_527_MOESM2_ESM.tif]

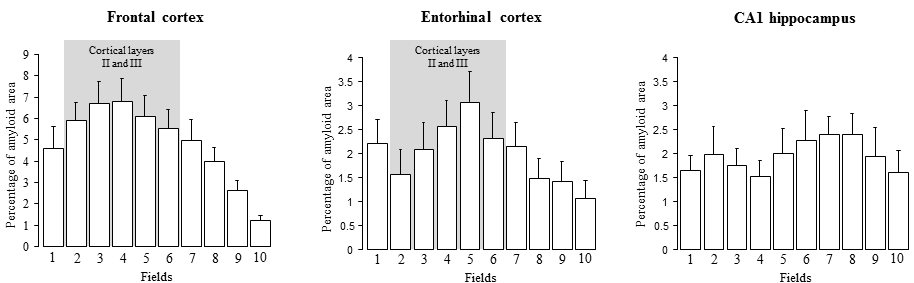

Supplement: Supplementary file 3 — Percentage of amyloid area according to fields. This percentage was calculated on the whole population of 25 cases. Field 1 corresponded to the cortex immediately under the pial surface and field 10 reached the white matter. Due to the poor representativeness of fields 1 (non tissular zone and pial surface) and 10 (proximal white matter), they were not included in statistical analysis for the cortical areas. The distribution of cortical layers was consistent with previously reported morphological studies ([18]; [17]). For instance, in frontal and entorhinal cortices, the cortical layer I was principally found in fields 1 and 2, cortical layers II and III were mostly represented in fields 2 to 6, layer IV was confined in fields 6 to 8, and layers V and VI were found in fields 7 to 10. Moreover, the Aβ deposits were more frequent in cortical layers II and III. As the fields were examined at a magnification of × 400, each field was 300 μM × 150 μM in size. (TIFF 35 kb) [file 40478_2018_527_MOESM3_ESM.tif]
